# The dynamics of the manifestations of atopic dermatitis in severe persistent uncontrolled asthma children with omalizumab therapy

**DOI:** 10.1186/2045-7022-5-S1-P3

**Published:** 2015-03-11

**Authors:** Elena Vishneva, Leyla Namazova-Baranova, Anna Alekseeva, Kamilla Efendieva, Anna Tomilova, Natalia Voznesenskaya, Konstantin Volkov, Julia Levina

**Affiliations:** 1 Scientific Center of Children Health, SRI of Preventive Pediatrics and Reabilitation, Moscow, Russia

## Background

in children with severe persistent uncontrolled asthma atopic dermatitis (AD) is often characterized by persistent continually relapsing.

According to the approach ICON for children > 6 y o with severe uncontrolled asthma as an additional treatment from 3-4 step of therapy omalizumab (Omab) is recommended for use of. We report on the experience of the use of Omab in children with asthma and AD.

## Methods

Medical files of 64 children treated with Omab were analysed: 36% of patients have had a concomitant diagnosis of AD, 39.1% of them suffered of severe AD (SCORAD >60-70) at baseline.

All of the following data were recorded, at baseline and after 6, 12 and 24 mo of Omab therapy: values of SCORAD, duration of periods of remission and periods of exacerbation of AD, quality of life (on a scale CDLQI and ACQ), requirement to use of topical steroids and maintenance asthma therapy, PFTs values, symptom scores and control level (GINA, ACT).

## Results

the characteristics of children with uncontrolled severe asthma and persistent AD were: mean age of the beginning of Omab therapy – 11.8 y, 60% girls, mean SCORAD index 72 points, quality of life (questionnaire CDLQI – mean 15,2), the requirement of topical steroids was high (mean 6.2 g/day of therapy), the duration of periods of remission were extremely short and periods of exacerbation of AD were more than 280 days/y. Initial maintenance asthma therapy was ICS (mean daily dose: 750µg fluticasone) with LABA, asthma symptom control questionnaires (mean ACT: 7.5, mean ACQ 23), mean FEV1 76%, mean PFT 69.7%.

After prolonged Omab therapy following indices significantly improved: mean SCORAD 28, 17 and 10 points (after 6, 12 and 24 mo accordingly), mean CDLQI 9, 4, and 2.1; the use of topical steroids 2.9, 1.6 and 0.7 g/d, periods of AD remission were extended, asthma control (mean ACT: 19, mean ACQ 12), mean FEV1 96%.

The effectiveness of treatment was directly correlated with the duration of therapy.

## Conclusions

Omab therapy not only improves asthma control, it allows to reduce of the manifestations and rate of exacerbations of severe AD and has shown steroid saving effect.

